# The Need for a Translational Model of Childhood Maltreatment: From Research to Principles to Action

**DOI:** 10.32872/cpe.22213

**Published:** 2026-02-27

**Authors:** Pia Pechtel

**Affiliations:** 1 Department of Psychology, University of Exeter, Exeter, United Kingdom; 2 Child and Youth Advocacy Centre Kelowna, Kelowna, Canada

Over the past two decades, research on the impact of childhood maltreatment (CM) has expanded rapidly, revealing complex physiological, neurobiological, and psychological effects. This growth in research is warranted: in 2025, the World Health Organization reported that over half of all children worldwide – more than 1 billion – experience some form of maltreatment each year. Despite this growing understanding, few studies demonstrate how this research translates into clinical practice. Without a clear translational framework, research risks remaining isolated, clinical care fragmented, and policy impact limited. The result is a gap between what we know and what we do.

A Translational Model of Childhood Maltreatment (TMCM) therefore needs to go beyond summarizing evidence. One effective approach is to turn *research findings* into *guiding principles* that directly inform *actionable strategies* across clinical settings. While calls for such translational efforts are not new (e.g., Samson et al., 2024), clinical psychology is now uniquely positioned to respond. This editorial presents a Research-Principles-Action framework for children and youth, showing how a TMCM can guide frontline practice. Given space constraints, this contribution does not propose a comprehensive model but instead serves as a call for action.

## TMCM: Research

Among the many insights from past research, five key findings stand out.

1. **CM Impacts Brain Development and Increases Vulnerability to Psychopathology** (e.g., Teicher & Samson, 2016; McLaughlin et al., 2019). CM is associated with changes in structural and functional brain development, partly through dysregulation of the hypothalamic-pituitary adrenal (HPA) axis and corticotropin-releasing factor (CRF) signalling. Neuroimaging studies have linked CM to altered threat detection, emotion reactivity and regulation, and reward processing. While these adaptations may have been protective in an abusive or neglectful environment, in safe contexts they can increase risk for psychopathology, including posttraumatic stress disorder (PTSD), major depression, and anxiety disorders.
2. **Type and Timing of CM Shape Clinical Trajectories and Brain Aging** (e.g., Fleming et al., 2025). Dimensions of CM (e.g., threat vs. deprivation), specific CM subtypes, and developmental timing of CM exposure are associated with distinct neurodevelopmental outcomes, clinical presentations, and acceleration in brain aging. CM represents a complex interaction of CM-related factors rather than an isolated experience.
3. **CM-Related Ecophenotype** (e.g., Teicher & Samson, 2013; Pechtel et al., 2022). Individuals sharing the same psychiatric diagnosis, with or without a history of CM, appear clinically and neurobiologically distinct. Those with CM histories often show altered HPA and CRF circuits, systemic inflammation, gene × environment interactions, and epigenetic changes. Clinically, they tend to present with an earlier disorder onset, higher comorbidity, greater symptom severity, and increased suicide risk. They also show reduced responsiveness to psychotherapy, pharmacological treatment, and combined treatment approaches, highlighting the need for tailored interventions to address unmet needs.
4. **Resilient Functioning Is Possible, but More Difficult to Achieve Following CM** (e.g., Ioannidis et al., 2020; McCrory et al., 2022). A range of resilience factors across individual, family, community, and neurobiological domains have been linked to “better-than-expected” outcomes following CM and can buffer against future stress. However, individuals exposed to CM are less likely to have these protective factors; for example, they often experience fewer supportive relationships (e.g., social thinning) and more frequent stressful interpersonal interactions (e.g., stress generation).
5. **Resilience Factor** × **Timing** (e.g., Filetti et al., 2024). Infancy and peripuberty are proposed as sensitive periods for the calibration and recalibration of stress-response systems, including the HPA axis and prefrontal-limbic circuitry. During these periods of heightened neuroplasticity, resilience factors (e.g., supportive caregiving, high-quality peer relationships, emotion regulation scaffolding) may become embedded in stress-regulatory processes and promote adaptive functioning.

## TMCM: Principles and Actions

Research indicates that TMCM’s guiding principles and actionable strategies must account for the complex effects of CM on the brain, behaviour, and clinical outcomes. Findings also support targeted, multidisciplinary interventions that take advantage of sensitive periods and help build resilience.

### P1. Mechanism-Informed Practice: Address Complexity (Findings 1-4)

Focusing on underlying neurobiological and psychological processes, rather than only on symptoms, allows interventions to be more tailored and effective. 

**Actionable Strategies:**
- Assess transdiagnostic mechanisms (e.g., emotion regulation, stress-response systems).
- Use clinical formulations to guide intervention selection, sequencing, and intensity.
- Implement multidisciplinary care (e.g., psychological, medical, community, and resource-based supports) to address broader needs arising from CM.
- Deliver integrated treatment that reduces psychopathology while promoting resilience across multiple domains (e.g., individual, family, community, school).

### P2. Timing Optimization: Consider Sensitive Periods (Findings 2 & 5)

Neurodevelopmental stages matter both at the time of CM exposure and at the time of treatment delivery, due to windows of heightened neuroplasticity. Evidence of accelerated brain aging following CM underscores the importance of timely interventions that harness sensitive periods. 

**Actionable Strategies:**
- Use formulation-based assessments that consider the timing of CM exposure and the child’s developmental stage.
- Deliver timely interventions optimized for early childhood and peripuberty to harness neuroplasticity.
- Monitor and adjust support based on developmental stage and brain aging.
- Conduct clinical research trials to examine optimal timing for interventions.

### P3. Integrated Approach: Address Psychopathology and Promote Resilience (Findings 1, 4 & 5)

An integrated approach addresses the psychological effects of CM while also promoting resilience across individual, family, and community levels. 

**Actionable Strategies:**
- Implement evidence-based interventions to address psychopathology (e.g., Trauma-Focused Cognitive Behavioural Therapy for PTSD) while promoting resilience (e.g., prosocial community activities that enhance social connectedness and belonging, positive parenting training to foster emotion regulation, rewarding experiences to buffer stress responses).
- Monitor symptoms, mechanisms, and resilience factors over time to guide formulation updates and multi-level interventions.
- Use formulation to coordinate support across care systems (e.g., social services, education, healthcare) to reduce systemic fragmentation.

**Figure 1:**
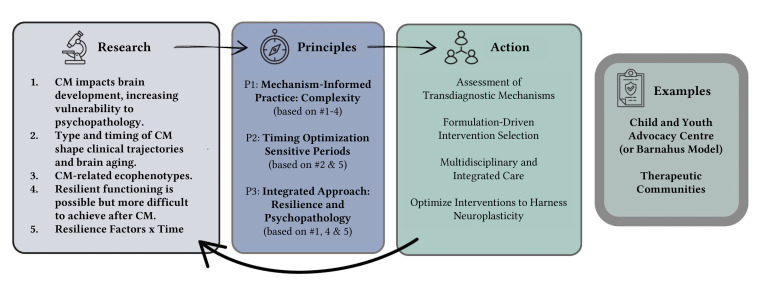
Translational Model of Childhood Maltreatment (TMCM): Research-Principles-Action

Figure 1 illustrates how key research findings on childhood maltreatment (CM) can be translated into guiding principles that inform actionable strategies. It also provides examples of two clinical settings where TMCM has been applied, highlighting the translation from research to the clinical frontline.

## Clinical Examples

Two clinical settings illustrate how the TMCM could translate into frontline practice.

### Child and Youth Advocacy Centre (CYAC)

Originating in the US in the mid-1980’s, CYACs now number over 1,000 centres across North America, with adapted models implemented across Europe. Their shared goal is to improve responses to CM through a multidisciplinary model that reduces systemic fragmentation and delivers integrated care. At the CYAC Kelowna in British Columbia, professionals from law enforcement, social services, victim support, education, healthcare, mental health, cultural services, and family advocacy all work together under one roof. Families receive formulation-based assessments to identify transdiagnostic mechanisms, followed by multi-level support tailored to the families’ unique needs (P1: *Mechanism-Informed Practice*). Treatment plans equally address psychopathology through evidence-based practice (e.g., Trauma-Focused Cognitive Behavioural Therapy for PTSD) and connect youth and families to resilience-promoting factors (e.g., high-quality peer connection, supportive parenting groups, prosocial activities to increase self-esteem; P3: *Integrated Approach*). Importantly, children, youth, and families access the CYAC directly after reports to child protection authorities, ensuring timely support. Support is then adapted to developmental needs from childhood through to adulthood (P2: *Timing Optimization*). Finally, formulations are shared across care systems (e.g., social services, health care, education, victim services) to ensure continuity of care (P3: *Integrated Approach)*.

### Therapeutic Community (TC)

TCs (De Leon & Unterrainer, 2020) are structured, living-learning programs where the community itself fosters resilience through social connectedness and supportive relationships. Participants also receive real-time support targeting transdiagnostic mechanisms, such as emotion regulation (P1: *Mechanism-Informed Practice*), while developing skills that promote growth and long-term resilience, including life and social skills (P3: *Integrated Approach*). A key strength of TCs is their ability to provide intensive programs during early childhood (e.g., mother-infant) and adolescence, aligning with sensitive periods of neuroplasticity (P2: *Timing Optimization*). Next-generation TCs aim to formally integrate evidence-based practices to address psychopathology within the lived, resilience-promoting environment.

## Concluding Thoughts

The TMCM provides a roadmap for translating research on CM into principle-driven, actionable strategies that guide clinical practice. Importantly, it does not replace the need to evaluate the effectiveness of these strategies across different contexts. In the TMCM framework, the evidence base serves as both the starting point and the end point, creating a system that evolves as knowledge grows. Incorporating new, robust findings ensures guiding principles remain actionable and scientifically grounded. Over time, this approach can shape policy and help ensure that children and families have access to the best possible support. The science is ready. The need is urgent. Now is the time to turn knowledge into action.
